# Clinical and molecular characterization of 12 prenatal cases of Cri‐du‐chat syndrome

**DOI:** 10.1002/mgg3.1312

**Published:** 2020-06-04

**Authors:** Ying Peng, Jialun Pang, Jiancheng Hu, Zhengjun Jia, Hui Xi, Na Ma, Shuting Yang, Jing Liu, Xiaoliang Huang, Chengyuan Tang, Hua Wang

**Affiliations:** ^1^ Department of Medical Genetics Hunan Provincial Maternal and Child Health Care Hospital Changsha Hunan China; ^2^ National Health Commission Key Laboratory of Birth Defects Research, Prevention and Treatment Changsha Hunan China; ^3^ Department of Nephrology Hunan Key Laboratory of Kidney Disease and Blood Purification The Second Xiangya Hospital Central South University Changsha Hunan China

**Keywords:** 5p deletion, Cri‐du‐chat syndrome, prenatal diagnosis, single nucleotide polymorphism array

## Abstract

**Background:**

This study aimed to define the molecular basis for 12 prenatal cases of Cri‐du‐chat syndrome (CdCS) and the potential genotyping‐phenotyping association.

**Methods:**

Karyotyping and single nucleotide polymorphism array analyses for copy number variants were performed.

**Results:**

Nine cases had 5p terminal deletions and three had 5p interstitial deletions, and these cases had variable deletion sizes with partial overlapping. Phenotypically, besides intrauterine growth restriction (IUGR) and brain as well as heart abnormalities, hypospadias, and lung dysplasia were observed. Potential genetic causes for specific phenotypes in these cases were identified.

**Conclusion:**

This study defined the molecular bases for the patients of CdCS, which is important for genetic counseling for these families. The findings of present study expand the clinical features of CdCS in the fetal period, and provided important information for further refining the genotypic–phenotypic correlations for this syndrome.

## Graphical abstract

This study provides clinical and molecular characterization for 12 prenatal case of Cri‐du‐chat syndrome (CdCS) and genotyping‐phenotyping analysis for these cases. The findings from the present study are important for genetic counseling for the families of affected individuals, expand the clinical features of CdCS in the fetal period, and provide important information for further refining the genotypic–phenotypic correlations for this syndrome.

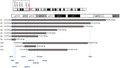

## 1 INTRODUCTION

Disorder resulting from a deletion of the short (p) arm of chromosome 5 is termed Cri‐du‐chat syndrome (CdCS) (OMIM#123450) or 5p deletion syndrome. CdCS is one of the most common chromosomal defect syndromes. It is estimated that CdCS occurs in 1 in 15,000–50,000 live birth infants (Niebuhr, 1978). Clinically, CdCS is typically characterized by high‐pitched cry (cat’s cry), microcephaly, low birth weight, and hypotonia in infancy (Kondoh et al., 2005; Mainardi et al., 2001). Other features, including intellectual disability, distinctive facial features, congenital heart defect (CHD), and behavioral problems have also been noticed in affected individuals (Elmakky et al., 2014; Nguyen et al., 2015).

Recent studies have associated some critical regions of 5p with the clinical features of this disorder including haploinsufficiency of 5p15.3 for cat‐like cry and speech delay, and haploinsufficiency of 5p15.2 for facial dysmorphism, microcephaly, and severe intellectual disability (Correa, Feltes, & Riegel, 2019; Zhang et al., 2005). Moreover, haploinsufficient deletions of some candidate genes in 5p have been associated with the specific clinical features of this disorder (Kondoh et al., 2005; Nguyen et al., 2015). For instance, haploinsufficiency of catenin delta 1 (*CTNND2*; OMIM *604275*)* and semaphorin 5A (*SEMA5A*; OMIM *609297) in 5p have linked to severe intellectual disability in individual affected with CdCS (Correa et al., 2019; Medina, Marinescu, Overhauser, & Kosik, 2000; Nguyen et al., 2015). Despite the progress in the clinical and molecular delineation of CdCS, the genotyping‐phenotyping association of CdCS remains largely unclear.

In this study, we defined the molecular bases for 12 prenatal cases of CdCS, and further identified that haploinsufficiency of iroquois homeobox 4 (*IRX4*; OMIM *606199), NADH‐ubiquinone oxidoreductase subunits 6 (*NDUFS6*; OMIM*603848), steroid 5 alpha‐reductase 1 (*SRD5A1*; OMIM*184753), and/or ADAM metallopeptidase with thrombospondin type 1 motif 16 (*ADAMTS16*; OMIM*607510) are associated with some specific phenotypes in CdCS.

## 2 MATERIALS AND METHODS

### 2.1 Ethical compliance

The study was approved by the ethics committee of Hunan Provincial Maternity and Infant Care Hospital.

### 2.2 Subjects

Twelve prenatal cases of CdCS that were collected in Prenatal Diagnosis Central of Hunan Provincial Maternity and Infant Care Hospital were included in this study. A brief introduction of the conditions of the cases and their parents are presented in Table 1. The reasons for being referred to our prenatal diagnosis central including ultrasound abnormalities in six cases, high risk of fetal aneuploidy identified by maternal serum screening test or noninvasive prenatal testing (NIPT) in four cases, positive family history for genetic diseases in one case, and the history of miscarriages in one case. Following written informed consents, chorionic villus (CVS), amniocentesis cells, and cord blood were collected for molecular analysis for the fetuses, and peripheral blood was collected from parents to determine inheritance patterns of any deletions identified.

**TABLE 1:** Information about the 12 fetuses with 5p deletion

Case	1	2	3	4	5	6	7	8	9	10	11	12
Mother age	26 years	27 years	27 years	30 years	28 years	34 years	27 years	41 years	25 years	31 years	24 years	35 years
Gestation history	G_3_P_0_	G_1_P_0_	G_1_P_0_	G_4_P_1_	G_2_P_1_	G_3_P_1_	G_1_P_0_	G_3_P_0_	G_1_P_0_	G_3_P_1_	G_3_P_0_	G_4_P_1_
Fetus age	10 weeks	30 weeks	36 weeks	24 weeks	35 weeks	17 weeks	26 weeks	19 weeks	23 weeks	32 weeks	22 weeks	21 weeks
Fetus sex	F	F	M	F	F	M	M	F	F	M	F	M
Fetus sample	CVS	UCB	UCB	AF	UCB	AF	AF	AF	AF	UCB	AF	AF
Reason for ascertainment	Miscarriage histor years	UT (+)	UT (+)	UT (+)	UT (+)	Mental retardation of the mother	UT (+)	NIPT (+)	NIPT (+)	UT (+)	MSS (+)	NIPT (+)

Abbreviations: AF, amniotic fluid; CVS, chorionic villus; F, female; G, gestation; M, male; MSS (+), maternal serum screening indicates high risk of trisomy 21; NIPT(+), genetic lesions by noninvasive prenatal testing; P, parturition; UCB, umbilical cord blood; UT (+), abnormalities on ultrasound testing.

### 2.3 Karyotyping and single nucleotide polymorphism array analyses

Karyotyping via routine chromosome G‐banded (320–400 bands) analyses were performed in cells in metaphase according to standard protocols (Peng et al., 2015). For single nucleotide polymorphism (SNP) array analysis, the genomic DNA was extracted from the CVS, amino fluid cells, cord blood, and peripheral blood lymphocytes by using DNA Isolation Kit for Cells and Tissues and QIAamp DNA Blood Mini Kit (QIAGEN), respectively. SNP array analysis was conducted using Affymetrix CytoScan^®^750 K Array (Affymetrix Inc) according to the manufacturer's instruction. Data from SNP array analysis was analyzed by using Chromosome Analysis Suite (ChAS; version 2.1). All genomic coordinates were taken from the human reference sequence. Genes and Online Mendelian Inheritance in Man (OMIM) references were from RefSeq and OMIM entries, respectively (Liu et al., 2016).

## 3 RESULT

### 3.1 Clinical findings

**FIGURE 1:**
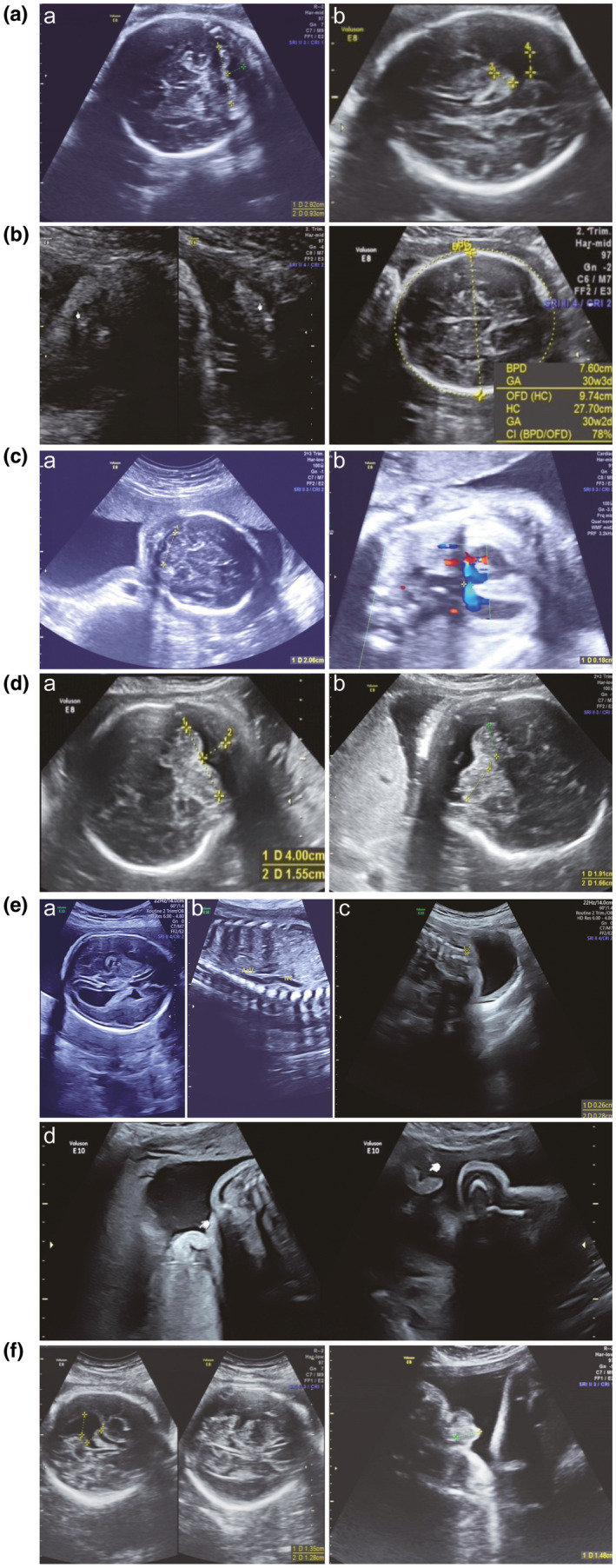
Images of ultrasound test on fetuses. A. Images of ultrasound test on fetus 2 at 30 weeks’ gestation. (a) Ultrasonographic image showing measurement of transverse cerebellar diameter (TCD). The TCD of this fetus was 2.92 cm. The normal value at 30 weeks’ gestation is 3.86 ± 0.34. The value of posterior fossa pool is 0.93 cm that is higher than the normal range (<0.80 cm). (b) Ultrasonographic image showing measurement of the left lateral ventricle. The highest value is 1.06 cm that is higher than the normal range (<0.80 cm). B. Images of ultrasound test on fetus 3 at 36 weeks’ gestation. (a) Ultrasonographic image showing that the lung tissue was scarce, suggesting lung dysplasia. (b) Ultrasonographic image showing measurement of biparietal diameter (BPD) and head circumference (HC). The value of BPD (7.6 cm) and HC (27.70 cm) represent 30 weeks’ gestation, suggesting an intrauterine growth retardation of fetus 3. C. Images of ultrasound test on fetus 4 at 24 weeks’ gestation. (a) Ultrasonographic image showing measurement of TCD. The TCD of fetus 4 was 2.06 cm. Normal value at 24 weeks’ gestation is 2.85 ± 0.17 cm. (b) Ultrasonographic image showing transventricular septal blood flow signal, suggesting ventricular septal defect. The ventricular septal defect sized 0.3 cm. D. Images of ultrasound test on fetus 5 at 35 weeks’ gestation. (a) Ultrasonographic image showing the TCD of this fetus was 4.00 cm. The normal value at 35 weeks’ gestation is 4.29 ± 0.26. The value of posterior fossa pool was 1.55 cm that is higher than the normal range (<0.80 cm). (b) Ultrasonographic image showing measurement of left and right cerebellar hemisphere, suggesting left cerebellar hemispheric dysplasia. E. Images of ultrasound test on fetus 7 at 26 weeks’ gestation. (a) Ultrasonographic image showing measurement of the left lateral ventricle. (b) Ultrasonographic image showing absence of inferior vena cava. (c) Ultrasonographic image showing neural tube defects. (d) Ultrasonographic image showing hypospadias. F. Images of ultrasound test on fetus 10 at 32 weeks’ gestation. (a) Ultrasonographic image showing measurement of the left lateral ventricle. The result of measurement was 1.28–1.35 cm. (b) Ultrasound image showing the coronal view of the external genitalia, suggesting hypospadias

Fetal ultrasound revealed that seven cases (case 1–5, 7, and 10) had developmental anomalies, and two cases (fetus 9, 11) showed no apparent abnormality. Results of fetal ultrasound for case 6, 8, and 12 were not found. Among those with abnormal ultrasound findings, three cases (1, 3, and 5) showed retarded embryo growth or intrauterine growth restriction (IUGR), five cases (2, 4, 5, 7, and 10) displayed cerebellar hypoplasia, and two cases (4 and 7) exhibited CHD. In addition, fetus 3 also had lung tissue dysplasia, and both fetus 7 and 10 showed hypospadias. The detail information regarding the clinical findings is listed in Table 2 and Figure 1.

**TABLE 2:** Ultrasound findings, and the results of karyotyping and SNP analysis

Case	Ultrasound findings	Karyotype	SNP array results			Parental Origin	Note
			Genomic coordinates (hg19) start–end	size (Mb)	Type		
1	Embryo stop growing	Not available	5p15.33p13.1 (113,576–39,068,326)	39.0	del	De novo	
2	Dysgenesis of the cerebellar, widened posterior fossa pool, and abnormality of lateral ventricle	46, XX, del (5) (p13)	5p15.33p13.3 (113,576–32,733,570)	32.6	del	De novo	
3	Intrauterine growth restriction, lung tissue dysplasia	46, XY, del (5) (p13)	5p15.33p13.3 (113,576–29,739,642)	29.6	del	De novo	
4	Ventricular septal defect, cerebellar hypoplasia	46, XX, del (5) (p14)	5p15.33p14.3 (113,576–19,255,769)	19.1	del	De novo	
5	Intrauterine growth restriction, left cerebellar hemispheric dysplasia, and widened posterior fossa pool	46, XX, del (5) (p15)	5p15.33p15.2 (113,576–14,738,108)	14.6	del	De novo	
6	Unavailable	46, XY, del (5) (p15)	5p15.33p15.2 (113,576–11,321,779)	11.2	del	mat	The mother had severe mental retardation
7	enlarged lateral ventricle, absence of inferior vena cava, neural tube defects, and hypospadias	46, XY, der(5) t(5;14) (p15.2; p32)mat	5p15.33p15.2 (113,576–10,459,497)	10.3	del	mat	The mother was normal
			14q31.3q32.33 (85,163,601–107,284,437)	22.1	dup		
8	Unavailable	46, XX, der (5)t(5;8) (p15.3; p12) mat	5p15.33 (113,576–3,923,103)	3.8	del	mat	The mother was normal
			8p23.3p12 (158,048–29,188,560)	29.0	dup		
9	Normal	46,XX	5p15.33 (113,576–2,035,548)	1.9	del	mat	The mother was normal
10	Enlargement of the lateral ventricle, hypospadias	46, XY, del (5) (p13)	5p15.32p13.3 (4,538,650–32,005,542)	27.5	del	De novo	
11	Normal	46, XX	5p15.32‐p15.2 (5,262,861–10,532,502)	5.2	del	pat	The father had mild intellectual disability
12	Unavailable	46, XY, del (5) (p14)	5p15.31p14.2 (7,116,243–24,560,212)	17.4	del	De novo	

Abbreviations: Mb, megabase pair; SNP, single‐nucleotide polymorphism.

Although fetus 11 appeared normal at 22 weeks of gestation by ultrasound testing, medical examination demonstrated that the father who carried the same 5p deletion had slight dysmorphic faces and mild intellectual disability. In addition, according to family history information, the first pregnancy of the same parents of fetus 11 was aborted because of the findings of CHD and diaphragmatic hernia at 7‐months gestation, and the second pregnancy was miscarried in the early stage.

### 3.2 Results of karyotyping and SNP array analyses

Karyotyping by G‐banding were performed for 11 fetuses (2–12), and revealed that 9 of them except for fetus 9 and 11 showed 5p deletions. In addition, in accompany with the 5p deletion, duplication of 14q31.3‐q32.33 and 8p23.3‐p12 were detected in fetus 7 and fetus 8, respectively. Fetus 7 and 8 inherited the 5p deletion from the unaffected mother who underwent a balanced chromosome translocation between chromosome 5 and other chromosome. The karyotyping results are listed in Table 2.

Single nucleotide polymorphism array analysis identified 5p deletions in all 12 fetuses, and mapped the breakpoints for the deletion fragments. Nine cases had terminal deletions starting at 113,576 base pairs (bp) (position of the first probe from the used BeadChip in 5p) with distinct end breakpoints between 2,035,54 bp and 39,068,326 bp, and three cases had interstitial deletions. The smallest deletion encompassed 1.9 Mb and the largest spanned 39.0 Mb. The breakpoints for the duplicated fragment in fetus 7 and 8 were also mapped. SNP array analysis for the parents of the fetuses revealed that the 5p deletions in seven fetuses (fetus 1–5, 10, 12) were de novo, and in another five fetuses were inherited. The results of SNP array analysis are listed in Table 2.

### 3.3 Haploinsufficiency of OMIM genes

**FIGURE 2:**
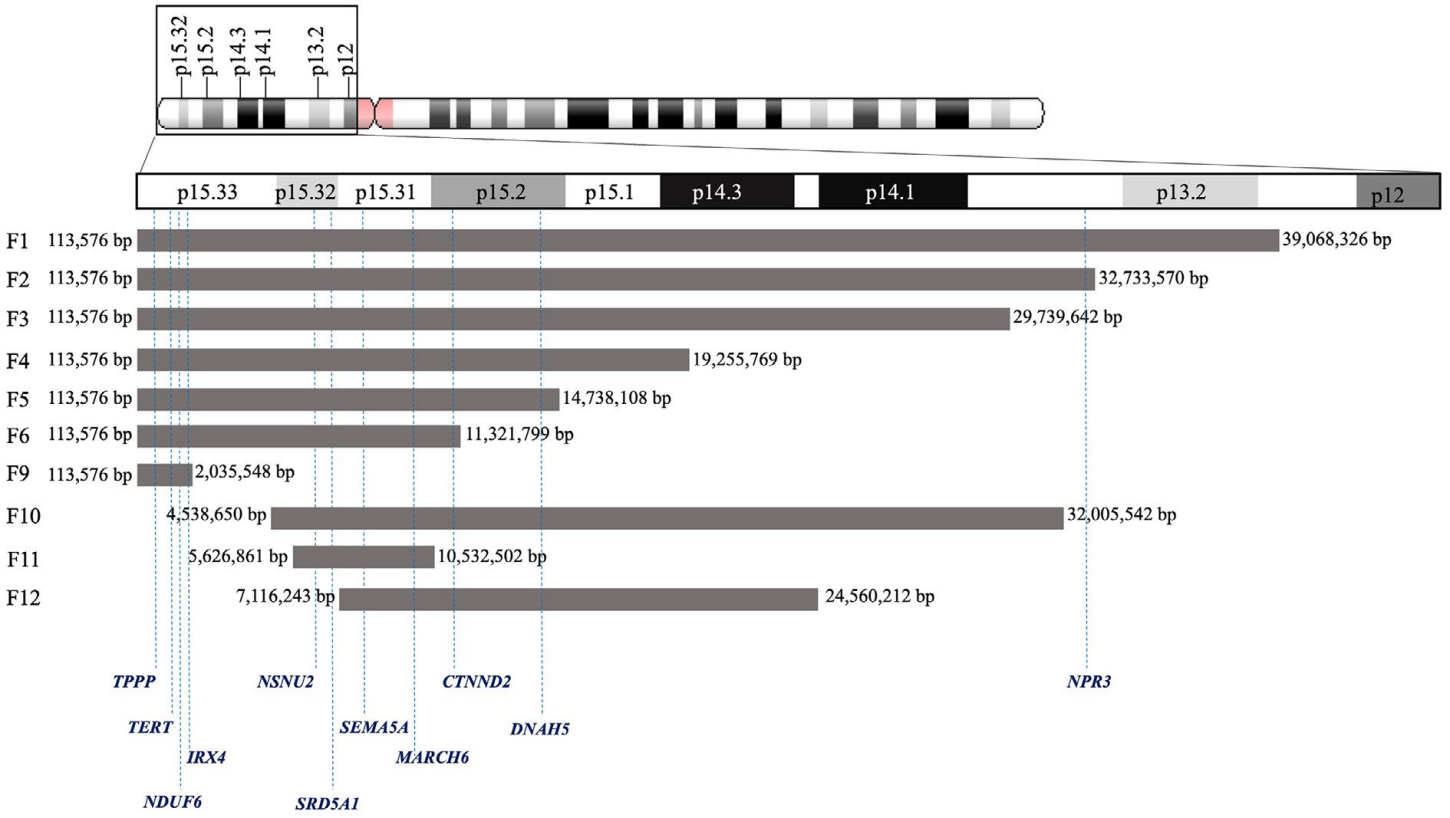
A comparison of the deleted regions and OMIM genes among the 5p deletion cases. OMIM, Online Mendelian Inheritance in Man

Recent studies have linked the haploinsufficiency of tubulin polymerization promoting protein (*TPPP*; OMIM*608773), telomerase reverse transcriptase (*TERT*; OMIM*187270), *IRX4*, *NDUFS6*, *ADAMTS16*, *CTNND2,* NOP2/Sun RNA methyltransferase 2 (*NSUN2*; OMIM*610916), *SEMA5A*, membrane associated ring‐CH‐type finger 6 (*MARCH6*; OMIM*613297), dynein axonemal heavy chain 5 (*DNAH5*; OMIM*603335), and natriuretic peptide receptor C (*NPR3*; OMIM*108962) among others to some specific feature of CdCS (Correa et al., 2019; Nelson, Jin, Downs, Kamp, & Lyons, 2014; Tan et al., 2016). Based on the information of the breakpoints for each deletion and the results of in silico analysis by using the UCSC Genome Browser Database and OMIM, OMIM genes in each deletion fragments were analyzed. The OMIM genes deleted in these fetuses are indicated in Figure 2.

## 4 DISCUSSION

Cri‐du‐chat syndrome was first identified in three patients by Lejeune in 1963 (Lejeune et al., 1963). Followed studies defined the typical clinical features of CdCS in adolescent and adult patients, including high‐pitched cry, microcephaly, speech delay, and intellectual disability, which are present with variable frequency (Overhauser et al., 1994; Zhang et al., 2005). To date, less than 50 prenatal cases of CdCS had been reported (Mak et al., 2019; Su et al., 2019). Furthermore, specific symptoms of CdCS during fetus period remain largely unknown. From July 2015 to December 2019, 12 cases of CdCS were identified in 28,564 pregnant women that were referred to our center to perform invasive prenatal diagnosis because of abnormal findings from fetal ultrasound testing and/or maternal serum screening, resulting in an incidence of 0.042% (12/28,564), which was similar to that reported by Su et al. (2019). Among them, nine cases had terminal deletions and three had interstitial deletions at 5p. The deletions in seven cases was de novo, while was inherited in other cases. The ratio of de novo 5p deletion in present study (58.3%) is lower than the previously reported (around 80%) (Mainardi et al., 2001). Phenotypically, among the nine cases with prenatal ultrasound results, seven cases had developmental anomalies, and two fetuses appeared normal. Among those with abnormal ultrasound findings, three had retarded embryo growth or IUGR, five had cerebellar hypoplasia, and two showed CHD. Hypospadias were noticed in two cases, and lung tissue dysplasia was detected in one case.

Abnormal brain development and function are one typical feature of CdCS patients in postnatal (Honjo et al., 2018; Zhang et al., 2005). In 36 prenatal cases of CdCS previously reported, 16 showed cerebral abnormalities such as cerebellar hypoplasia and abnormality of the cerebral ventricles (Mak et al., 2019; Su et al., 2019). In the present study, five fetuses (2, 4, 5, 7, and 10) showed abnormal brain development (Figure 1A,C–F). Notably, besides the 5p deletion, case 7 also contained a 22.1 Mb duplication of 14q31.3‐q32.33. In patients with 14q distal trisomy, phenotypes including growth retardation, hypertelorism, facial dysmorphia, and cardiovascular anomalies have been reported (Villa et al., 2016), but not neural tube defect and hypospadias. Therefore, neural tube defect and hypospadias in case 7 was most likely from the 5p deletion. Although fetus 6 and 11 showed no brain anomaly by fetal ultrasound, the parent who carried the same chromosomal abnormalities showed intellectual disability, suggesting haploinsufficiency of the two regions were potentially associated with brain development. The absences of brain anomaly in fetus 6 and 11 may arise from incomplete penetrance. Of note, the products of *TPPP, NSUN2,* and *SEMA5A* that localize in 5p13.3 have been demonstrated to play major roles in brain development (Blanco et al., 2014; Lehotzky et al., 2010; Mosca‐Boidron et al., 2016; Sardina, Walters, Singh, Owen, & Kimonis, 2014). *CTNND2* that localize in 5p13.2 have also been linked with brain development (Hofmeister et al., 2015; van Rootselaar et al., 2017). Moreover, fetuses 2, 4, 5, 6, 7, 10, and 11 contained an overlapping deletion region of 5p15.33‐p15.2 (position: 5,626,861–10,495,497) that contained *NSUN2* and *SEMA5A* (Figure 2). Together, these finding suggest that haploinsufficiency of 5p15.33‐p15.2 contributes critically to the abnormal brain development and mental retardation in patients of CdCS.

CHD has been reported in 15%–20% of patients of CdCS (Hills, Moller, Finkelstein, Lohr, & Schimmenti, 2006; Nagy et al., 2019; Zhu et al., 2016). The cardiac anomaly in CdCS includes patent ductus arteriosus, septal defects, Tetralogy of Fallot (TOF), and other structural malformations (Hills et al., 2006). In present study, fetus 4 that contained a deletion of 5p15.33‐p14.3 (position: 113,576–19,255,769) displayed ventricular septal defect (Figure 1C). Fetus 7 that contained a deletion of 5p15.33p15.2 (position: 113,576–10,459,497) showed the absence of inferior vena cava (IVC) (Figure 1E). These two fetuses shared an overlapping region from position 113,576 to 10,459,497. Proteins encoded by *IRX4*, *NDUFS6*, and *DNAH5* that localize in this regions have been demonstrated to be critical for cardiovascular development (Nelson et al., 2016; Nothe‐Menchen et al., 2019; Rouzier et al., 2019). The deletion region in fetus 4 contained these three region, and the deletion region in fetus 7 contained *IRX4* and *NDUFS6,* suggesting that haploinsufficiency of *IRX4* and/or *NDUFS6* lead to CHD.

Hypospadias were rarely reported in patients of CdCS. Chen et al. reported a prenatal case with distal deletion involving 5p15.1 → pter that displayed cerebellar hypoplasia, hypospadias, and facial dysmorphisms (Chen et al., 2013). In the present study, two fetuses (7 and 10) showed hypospadias (Figure 1E,F). Fetus 7 contained a deletion of 5p15.33‐p15.2 (position: 113,576–10,459,497), and fetus 10 carried a deletion of 5p15.32‐p13.3 (position: 4,538,650–32,005,542). Among the OMIM genes in their overlapping deleted region, haploinsufficiency of *SRD5A1* has been implicated as a candidate genetic reason for hypospadias. Notably, another two studies provided evidence that mutations of *SRD5A2*, instead of *SRD5A1*, were present in some boys with isolated hypospadias (Sun, Zhou, & Liu, 2019; Yuan et al., 2017). *SRD5A1* and *SRD5A2* encode isoform 1 and 2 of 5α‐reductase that catalyzes the conversion of testosterone into the more potent androgen, dihydrotestosterone, and their protein products shared 50% sequence identity, but whether hemizygous deficiency each of them have similar effect in the development of hypospadias awaits future functional analysis.

In the present study, we report a case of CdCS showing lung dysplasia of fetus 3 (Figure 1B). The fetus 3 contained a deletion of 5p15.33‐p13.3 (position: 113,576–29,739,642), which contained two OMIM genes *DNAH5* and *ADAMTS16* were the deletion region. Li et al. demonstrated that the protein production of *DNAH5* played an important role in the development of lung (Li et al., 2016). Emerging evidence also suggested that ADAMTS16 expressed at high levels in fetal lung, but its involvement in lung development remain unknown (Surridge et al., 2009).

5p deletions with no apparent phenotype have been reported (Nguyen et al., 2015). Similar 5p deletions were also reported in individuals with/without clinical phenotypes (Nguyen et al., 2015). In this study, fetus 11 containing a deletion of 5p15.32‐p15.2 (position: 5,262,861–10,532,502) showed no developmental abnormality by ultrasound testing. As such, the parents decided to continue the pregnancy, and a girl was born at the 39 weeks’ gestation. Physical examination showed the newborn had birth weight of 3,200 g (75th–90th), head circumference of 31.5 cm (10th–25th), and length of 45 cm (~50th). An Apgar score was 9 and 10 at the 1st and 5th minute after birth. Postoperative follow‐up, feeding, and physical examination showed that the child was normal. In addition, results of development assessment, including cognitive aspects, language, mobility, and dexterity were all normal. In addition, fetus 9 that inherited a 1.9 Mb terminal 5p deletion from a healthy mother showed no developmental abnormality by ultrasound testing at the gestation of 23+ weeks. The reasons for the absence of clinical phenotypes in case 11 and 9 remain largely unknown, but in completed penetration may be a potential reason.

5p deletions mostly arise de novo, and 80%–90% of which are paternal in origin potentially due to breakage of chromosome 5 during gamete formation in males (Campbell et al., 2014; Eyal et al., 2019). Around 15% of 5p deletions are from parental translocation between chromosome 5 and others. Less common mechanisms including mosaicism, inversions, or ring chromosomes have also been implicated (Cerruti Mainardi, 2006). The risk of recurrence can be negligible for the cases of a de novo 5p deletion, but attention should be paid to the possibility of gonadal mosaicism in the parents which increases the risk of recurrence (Cerruti Mainardi, 2006). The risk of producing a CdCS offspring in the families with translocation involving 5p deletion ranged from 8.7% to 18.8% (Cerruti Mainardi, 2006). In the present study, the 5p deletions in case 7 and 8 were from the mother with balanced translocation involved in 5p deletion. Thus, genetic counseling is critically important for their families.

In summary, this study clarified the genetic diagnosis for 12 prenatal cases of CdCS from different families, which was important for guidance of the future pregnancy. In addition, this study provides more material for the clinical manifestation of CdCS in the fetal period, which is helpful for further analysis of the genotype‐phenotype correlation in CdCS. This study also provided evidence supporting the critical regions and candidate genes whose haploinsufficiency is causative for clinical features of CdCS.

## CONFLICT OF INTEREST

All authors declare that they have no commercial or other conflicting interests.

## AUTHOR CONTRIBUTION

Y Peng and H Wang designed the study. Y Peng and C Tang prepared the manuscript. J Pang and N Ma performed the SNP array analysis. Y Peng, J Liu, and S Yang interpreted the data of SNP array analysis. J Hu, H Xi, and Z Jia analyzed the karyotype study. X Huang performed the prenatal ultrasound testing.

## STATEMENT OF ETHICS

Written informed consent was obtained prior to investigation. The authors have no ethical conflicts to disclose.

## Data Availability

The data that support the findings of this study are available from the corresponding author upon reasonable request.
